# Arginine does not rescue p.Q188R mutation deleterious effect in classic galactosemia

**DOI:** 10.1186/s13023-018-0954-8

**Published:** 2018-11-26

**Authors:** Minela Haskovic, Britt Derks, Liesbeth van der Ploeg, Jorn Trommelen, Jean Nyakayiru, Luc J. C. van Loon, Sabrina Mackinnon, Wyatt W. Yue, Roy W. A. Peake, Li Zha, Didem Demirbas, Wanshu Qi, Xiaoping Huang, Gerard T. Berry, Jelle Achten, Jörgen Bierau, M. Estela Rubio-Gozalbo, Ana I. Coelho

**Affiliations:** 1 Department of Clinical Genetics, Maastricht University Medical Center +, Maastricht, The Netherlands; 2 Department of Pediatrics, Maastricht University Medical Center +, Maastricht, The Netherlands; 3 GROW-School for Oncology and Developmental Biology, University of Maastricht, Maastricht, The Netherlands; 4 Department of Dietetics, Maastricht University Medical Center +, Maastricht, The Netherlands; 5 Department of Human Biology, NUTRIM School of Nutrition and Translational Research in Metabolism, Maastricht University Medical Center +, Maastricht, The Netherlands; 6 Structural Genomics Consortium, Nuffield Department of Clinical Medicine, University of Oxford, Oxford, UK; 7 Department of Laboratory Medicine, Boston Children’s Hospital, Harvard Medical School, Boston, MA USA; 8 The Manton Center for Orphan Disease Research, Division of Genetics and Genomics, Boston Children’s Hospital, Harvard Medical School, Boston, MA USA

**Keywords:** Classic galactosemia, Inherited metabolic disorder, Galactose metabolism, Arginine, Amino acid supplementation, Chemical chaperones

## Abstract

**Background:**

Classic galactosemia is a rare genetic metabolic disease with an unmet treatment need. Current standard of care fails to prevent chronically-debilitating brain and gonadal complications.

Many mutations in the *GALT* gene responsible for classic galactosemia have been described to give rise to variants with conformational abnormalities. This pathogenic mechanism is highly amenable to a therapeutic strategy based on chemical/pharmacological chaperones. Arginine, a chemical chaperone, has shown beneficial effect in other inherited metabolic disorders, as well as in a prokaryotic model of classic galactosemia.

The p.Q188R mutation presents a high prevalence in the Caucasian population, making it a very clinically relevant mutation. This mutation gives rise to a protein with lower conformational stability and lower catalytic activity. The aim of this study is to assess the potential therapeutic role of arginine for this mutation.

**Methods:**

Arginine aspartate administration to four patients with the p.Q188R/p.Q188R mutation, in vitro studies with three fibroblast cell lines derived from classic galactosemia patients as well as recombinant protein experiments were used to evaluate the effect of arginine in galactose metabolism. This study has been registered at https://clinicaltrials.gov (NCT03580122) on 09 July 2018. Retrospectively registered.

**Results:**

Following a month of arginine administration, patients did not show a significant improvement of whole-body galactose oxidative capacity (*p* = 0.22), erythrocyte GALT activity (*p* = 0.87), urinary galactose (*p* = 0.52) and urinary galactitol levels (*p* = 0.41). Patients’ fibroblasts exposed to arginine did not show changes in GALT activity. Thermal shift analysis of recombinant p.Q188R GALT protein in the presence of arginine did not exhibit a positive effect.

**Conclusions:**

This short pilot study in four patients homozygous for the p.Q188R/p.Q188R mutation reveals that arginine has no potential therapeutic role for galactosemia patients homozygous for the p.Q188R mutation.

**Electronic supplementary material:**

The online version of this article (10.1186/s13023-018-0954-8) contains supplementary material, which is available to authorized users.

## Background

Classic galactosemia (CG) (OMIM #230400) is a rare metabolic disease caused by a severe deficiency of galactose-1-phosphate uridylyltransferase (GALT), the second enzyme of the main pathway for galactose metabolism. It is an autosomal recessive disorder with a prevalence of 1:16,000 to 1:50,000 live births in Western countries.

Classic galactosemia represents a high burden in the lives of patients and families [1]. Its current standard of care, a galactose-restricted diet, even though life-saving in the neonatal period, fails to prevent chronically-debilitating complications, such as brain, gonadal and social impairments. Brain complications result in cognitive, behavioral and neurological complications. Patients often achieve a lower grade of education and occupation, they are often shy and less often succeed in building a relationship outside the family. More than 80% of female patients suffer from primary ovarian insufficiency (POI) with subsequently subfertility [2, 3]. Therefore, new therapeutic strategies are needed.

Several mutations in the *GALT* gene have been described to give rise to variants with a severely impaired catalytic activity due to GALT misfolding and/or aggregation in yeast and bacterial (*E. coli*) models [4, 5]. Notably, variant p.Q188R presented a strikingly high aggregation propensity [5]. Recently, the crystal structure of human GALT was determined by McCorvie et al. [6], providing a molecular framework to understand disease-causing mutations at the protein level. The c.563A > G (p.Q188R) mutation, the most frequent *GALT* mutation in the Caucasian population (> 60 to > 90% prevalence) [7] was described to give raise to a protein with reduced conformational stability and catalytic activity [6].

A promising therapeutic approach for conformational disorders focuses on the use of chemical/pharmacological chaperones [8–10]. Arginine is a chemical chaperone described to act as a selective inhibitor of undesirable protein aggregation [11, 12].

In vitro studies using a prokaryotic model of classic galactosemia revealed that arginine might be of potential value for a number of clinically-relevant mutations, including p.Q188R [13].

Studies in fibroblasts from patients with a mild peroxisomal biogenesis disorder revealed that arginine exerted a positive effect on the peroxisome function [14]. More recently, Sorlin et al. described the case of a patient with PEX12 deficiency that showed a positive effect at the biochemical and neurophysiological levels following 12 months of treatment with arginine [15]. Additionally, administration of arginine aspartate (in the commercially available form of Asparten®) was reported to have positive clinical and biochemical effects in a patient with pyruvate dehydrogenase deficiency (PDHc) [16].

The aims of this study were to evaluate the potential therapeutic role of arginine in classic galactosemia caused by the p.Q188R mutation.

## Results

### Arginine aspartate supplementation in patients

Four classic galactosemia patients homozygous for the c.563A > G (p.Q188R) mutation were enrolled in the clinical study. Patients’ characteristics are presented in Table 1.

**Table 1:** Patients’ characteristics

	Patient 1	Patient 2	Patient 3	Patient 4
Age (years)	29	21	19	24
Gender	Male	Female	Male	Female
Ethnicity	Caucasian	Caucasian	Caucasian	Caucasian
Genotype	p.Q188R/p.Q188R	p.Q188R/p.Q188R	p.Q188R/p.Q188R	p.Q188R/p.Q188R

Patients’ characteristics of the four classic galactosemia patients enrolled in the clinical study

Assessments of the effect of arginine aspartate supplementation (Asparten®) included whole body galactose oxidative capacity (primary outcome), erythrocyte GALT activity, as well as urinary galactose and galactitol levels (secondary outcomes). Baseline results were obtained immediately before Asparten® supplementation. Post treatment results were obtained after 30 ± 5 days of Asparten® intervention, immediately after suspension of treatment.

**Fig. 1:**
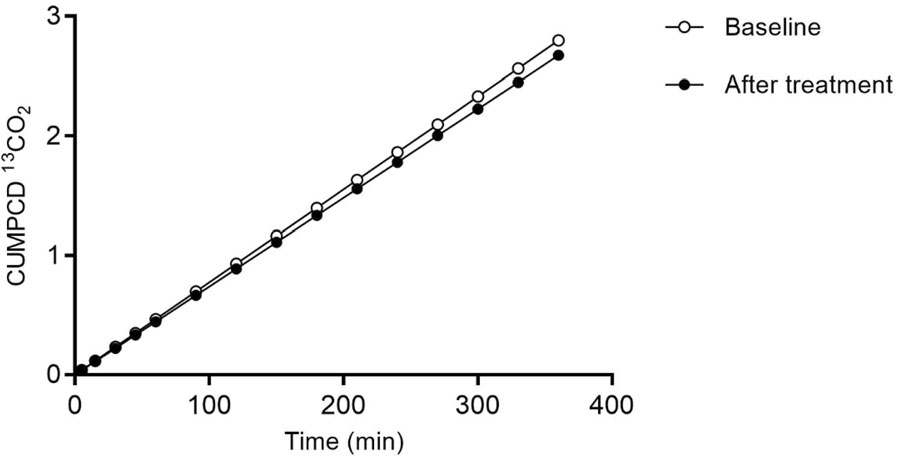
Mean galactose oxidative capacity before and after arginine aspartate supplementation. Mean galactose oxidative capacity of the four patients, expressed as CUMPCD (cumulative percent of the dose) ^13^CO_2_ eliminated in air. Baseline: CUMPCD (120 min) = 0.7 ± 0.06%; CUMPCD (360 min) = 2.8 ± 1.3%. After intervention: CUMPCD (120 min) = 0.7 ± 0.02%; CUMPCD (360 min) = 2.7 ± 1.2%

No significant difference in the profile of mean galactose oxidative capacity was observed (Fig. 1 and Additional file 1: Figure S1). The CUMPCD (cumulative percent of the dose) for the total time of the study (360 min) per patient is shown in Table 2. At baseline, the mean ^13^CO_2_ released by the four patients was 2.8 ± 1.3%, whereas after intervention it was 2.7 ± 1.2% (*p* = 0.22).

**Table 2:** Primary and secondary outcomes of the clinical study

	Patient 1		Patient 2		Patient 3		Patient 4	
	Baseline	After treatment	Baseline	After treatment	Baseline	After treatment	Baseline	After treatment
CUMPCD ^13^CO_2_ at 360 min (%)^a^	2.5	2.6	2.8	2.8	2.9	2.6	3.0	2.7
GALT activity (μmol/h/mmol Hb)	6.7 (1.2%)	6.1 (1.1%)	11.3 (2.0%)	16.0 (2.8%)	8.8 (1.5%)	5.7 (1.0%)	6.9 (1.2%)	6.9 (1.2%)
Galactitol in urine (μmol/mmol creatinine)	115	123	132	123	114	106	93	87
Galactose in urine (μmol/mmol creatinine)	6	11	5	6	8	5	24	7
Dietary arginine intake (g/day)	6	6	4	3	4	4	3	4
Compliance to Asparten ® (%)	100		93		92		98	
Days of treatment	28		28		35		35	

Supplementation of arginine aspartate (Asparten®) was evaluated by whole body galactose oxidative capacity (primary outcome), erythrocyte GALT activity, as well as urinary galactose and galactitol levels (secondary outcomes). Baseline evaluation was done immediately before the initiation of Asparten® supplementation, after treatment evaluation was done immediately after suspension of Asparten® supplementation

^a^ CUMPCD (cumulative percent of the dose) ^13^CO_2_ eliminated in air at 360 min

**Fig. 2:**
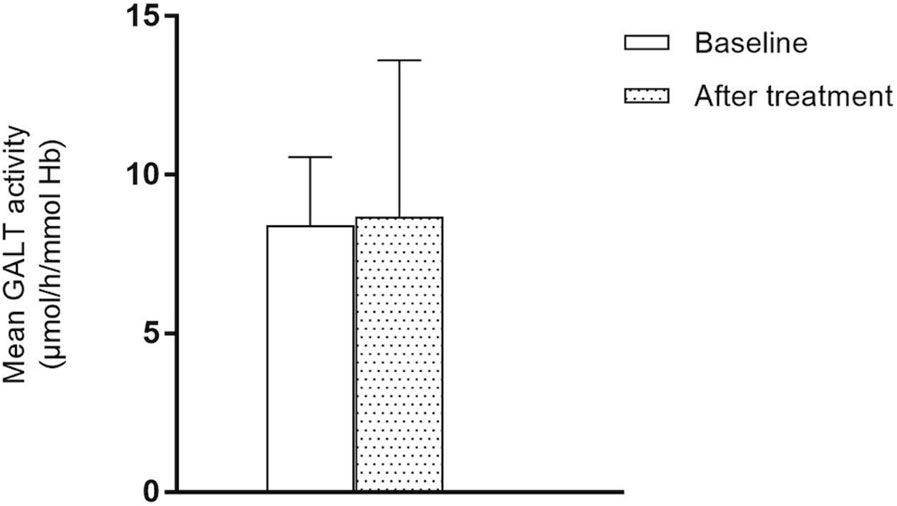
Mean GALT activity before and after arginine aspartate supplementation. GALT activity is expressed as μmol of UDP-Gal formed per hour per mmol hemoglobin

GALT activity before and after intervention is represented in Fig. 2. There was no statistically significant (*p* = 0.87) increase in GALT activity following arginine aspartate supplementation (baseline: 8.4 ± 2.2 μmol/h/mmol Hb, corresponding to 1.5%; after treatment: 8.7 ± 4.9 μmol/h/mmol Hb, corresponding to 1.5%) (Table 2).

**Fig. 3:**
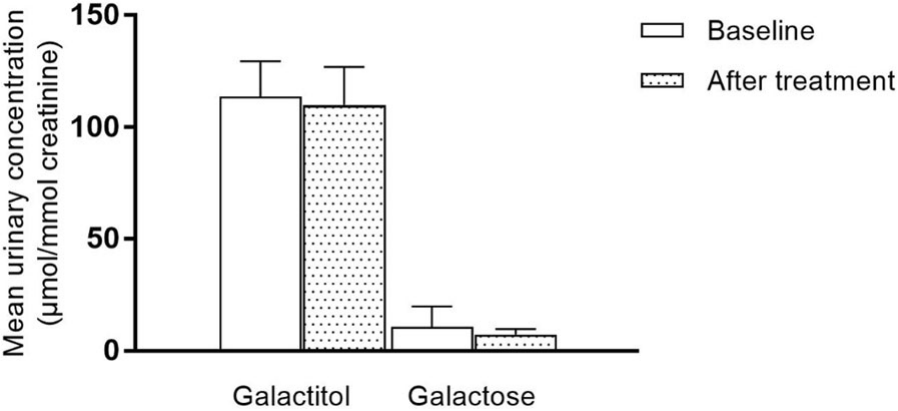
Mean urinary galactitol and galactose levels before and after arginine aspartate supplementation

Mean urinary galactitol and galactose levels are shown in Fig. 3. Both metabolites did not show a statistically significant decrease after arginine aspartate supplementation. Galactitol decreased from 114 ± 16 to 110 ± 17 μmol/mmol creatinine (*p* = 0.41) (reference 0–125 μmol/mmol creatinine) and galactose decreased from 11 ± 9 to 7 ± 3 μmol/mmol creatinine (*p* = 0.52) (Table 2).

Amino acid profile analysis revealed that plasma arginine decreased from 67 ± 17 μmol/L at baseline to 53 ± 21 μmol/L following intervention (*p* = 0.05) (reference 16–124.9 μmol/L). Additionally, patients exhibited on average an increase in plasma ornithine levels from 66 ± 25 μmol/mmol at baseline to 142 ± 73 μmol/L following intervention (*p* = 0.07) (reference 24.0–167.9 μmol/L). Other amino acids did not show any significant difference between baseline and after intervention.

The mean dietary arginine intake did not increase during study (4 ± 1 g/day at baseline and after treatment) (*p* = 0.87). Mean daily galactose intake did not change during study period. All patients showed a high compliance to Asparten® intake, ranging from 92 to 100% (Table 2).

Patient 1 reported headache in the first two days of Asparten® supplementation.

### Arginine exposure of patients’ fibroblasts

In parallel with the clinical analysis, fibroblasts derived from two p.Q188R/p.Q188R patients and one p.Q188R/p.Q188P patient were cultured in the presence and absence of arginine. GALT activity of exposed and unexposed fibroblasts is presented in Table 3. Patients’ fibroblasts displayed no detectable activity even in the presence of a high concentration of arginine (1 mM). In order to discard possible arginase activity, arginine in the cell culture medium was measured. Results are presented in Additional file 2: Table S1.

**Table 3:** GALT enzymatic activity in fibroblasts exposed to arginine

GALT activity (μmol/h/g protein)			
Cell line	No arginine	0.1 mM arginine	1 mM arginine
Control 1 (*n* = 3)	31.6 ± 12.9	45.6 ± 25.1	35.3 ± 21.3
Control 2 (*n* = 2)	52.9	46.1	42.9
CG 1 (n = 2)	n.d.	n.d.	n.d.
CG 2 (n = 2)	n.d.	n.d.	n.d.
CG 3 (n = 2)	n.d.	n.d.	n.d.

Two wildtype (controls 1 and 2) and three classic galactosemic fibroblasts derived from two p.Q188R/p.Q188R patients (cell lines CG1 and CG2) and a p.Q188R/p.Q188P patient (cell line CG3) were cultured in the absence and in the presence of supplemental arginine (0.1 mM and 1 mM arginine). Results are expressed as μmol per hour per gram protein and presented as mean ± SD or average for *n* = 2. Number of replicates is presented in brackets

n.d.: non-detectable

### Thermal shift assay of recombinant human GALT in the presence of arginine

**Fig. 4:**
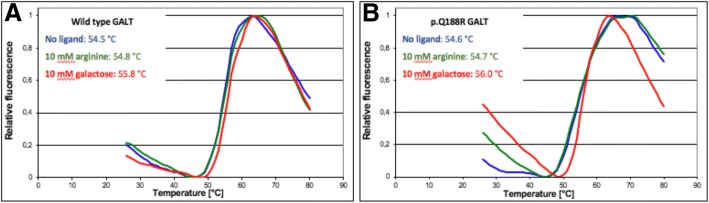
Representative thermal shift assay curves with no added ligand, arginine (10 mM) or galactose (10 mM) for A. wildtype GALT and B. p.Q188R GALT. Fluorescence values were normalized to allow comparison of Tm values. Curves for single replicate per ligand state plotted, with no added ligand, arginine (10 mM) or galactose (10 mM). Values stated are the mean values of three replicates

The therapeutic benefit of arginine was previously attributed to a possible role as chemical chaperone, stabilizing disease-associated proteins. To investigate this possibility, the in vitro thermal shift assay (differential scanning fluorimetry, DSF) was performed to evaluate whether arginine could thermally stabilize recombinant GALT (Table 4 and Fig. 4). Neither the p.Q188R or the wildtype GALT protein showed a thermal shift in the presence of arginine at any of the concentrations studied. In contrast, galactose (10 mM), a moiety of the substrate galactose-1-phosphate, caused a small shift in the melting temperature of the wildtype protein (ΔTm = 2.3 °C) but not of the p.Q188R variant (ΔTm = 1.4 °C).

**Table 4:** Thermal shift assay of wildtype and p.Q188R GALT

Melting temperature (Tm, °C)		
	Wildtype GALT	p.Q188R GALT
None	55.8 ± 0.2	54.6 ± 0.2
Arginine	54.8 ± 0.1	54.7 ± 0.4
Galactose	58.1 ± 0.4	56.0 ± 0.3

Comparison of temperature at which half the protein was unfolded (melting temperature, Tm) with no added ligand, arginine (10 mM) or galactose (10 mM). Values presented are the mean values of three replicates

## Discussion

In this study we evaluated the potential therapeutic role of arginine in classic galactosemia caused by c.563A > G (p.Q188R) mutation at three different levels: patients, fibroblast galactosemic cell lines and recombinant protein studies.

Patients were treated with arginine aspartate (in the commercially available form of Asparten®) in a dose of 15 g/day for one month. Arginine aspartate supplementation (Asparten®) did not enhance galactose oxidation rates or improve the biochemical profile in CG patients.

Whole body galactose oxidative capacity was evaluated for 6 h (360 min) after administrating a bolus injection of [1-^13^C]-galactose. The overall profile of galactose oxidative capacity did not show an increase following intervention (*p* = 0.22) (Fig. 1 and Additional file 1: Figure S1). All patients exhibited a CUMPCD less than 2% at 120, before and after treatment, consistent with a severe GALT deficiency, as described by Berry et al. [17].

GALT activity analysis in red blood cells (RBC) revealed no statistically significant difference after treatment compared to baseline. Galactose metabolite concentrations did not significantly change.

All patients continued with their galactose-restricted diet, the lifelong mainstay of treatment in classic galactosemia nowadays.

Amino acid profile analysis revealed that plasma arginine decreased whereas ornithine levels increased. It is known that arginase, the enzyme responsible for the conversion of arginine into ornithine, exhibits an increased transcription and activity upon exposure to exogenous arginine [18], which very likely accounted for the observed inverse plasmatic concentrations of these amino acids.

No adverse events have ever been reported since Asparten® entered in the market in 1974. In this study, arginine was well tolerated, but patient 1 reported headache in the first two days of Asparten® supplementation. This could possibly be due to vasodilation caused by nitric oxide production (NO) [19].

All patients showed a high compliance to Asparten® (93 to 100%) which suggests that the observed lack of efficacy was not due to low adherence to intervention. Dietary arginine intake was also evaluated due to its potential to influence results. At baseline, the arginine intake of patient 1 was nearly the double of that of the other patients (92% higher) and after treatment it was 123% of that of the other patients, which might suggest that increasing Asparten® administration would not improve the biochemical phenotype. It is important to keep in mind, however, that the metabolism of dietary arginine and Asparten® supplementation might differ, e.g. at the bioavailability level.

This is the first study to investigate the effect of arginine aspartate (Asparten®) supplementation in classic galactosemia. Asparten® has shown a positive effect in a PDHc deficient patient [16]. After 1 month of Asparten® administration, the 6-year-old patient showed a striking improvement on both biochemical and clinical outcomes. The patient was on a daily dose of 5 g arginine aspartate (Asparten®). In the present study, the adult patients were on a daily dose of 15 g arginine aspartate (Asparten®). However, Asparten® did not ameliorate the biochemical phenotype of the studied patients.

These findings contradict the previous study on the prokaryotic model of galactosemia, in which arginine supplementation to the medium in a concentration of 25 mM partially rescued the bacterial culture expressing human GALT p.Q188R [13]. These discrepancies might stem from the high arginine concentration used in the prokaryotic model.

Limitations of this study are the small number of subjects and relatively short duration of Asparten® supplementation. With respect to the RBC GALT activity, the lifespan of erythrocytes is about 120 days, both in galactosemic and in non-galactosemic patients. Since the study was conducted for 1 month, the effect of Asparten® on the GALT activity could be underestimated [20, 21]. The short duration of the study was also insufficient to evaluate Asparten® effects on long-term clinical outcomes. Furthermore, younger patients could have responded differently to Asparten®‘s treatment. However, neither fibroblast assays nor in vitro studies using recombinant human p.Q188R GALT showed a positive effect, which suggests that arginine’s mechanism of action as a chemical chaperone is not effective for this GALT variant.

These findings do not preclude that arginine could be successfully used in patients carrying other *GALT* mutations that lead to a conformational change without irreversibly affecting the catalytic activity. Further studies are warranted to gain more insight in arginine’s potential effect on other less frequent *GALT* mutations.

Recently, the crystallographic structure of human GALT has been reported [6], and will contribute for the development of GALT-based therapeutic approaches, namely pharmacological chaperones (PCs). PCs are a promising therapeutic approach in protein misfolding diseases. PCs bind specifically to the target protein and shift the equilibrium towards the correctly folded state of the protein. A number of pharmacological chaperones has been successfully developed in the field of inherited metabolic disorders (reviewed in ref. [10, 22]). Arginine is a chemical chaperone described to act as a selective inhibitor of undesirable protein aggregation [11, 12].

The new knowledge and understanding gained with the human GALT crystal structure holds great promise for the development of a PC-based therapeutic approach for CG. Furthermore, the crystal structure will also guide other developments of therapies for this disease, besides the PC-based approach. Other therapeutic approaches include substrate reduction therapies by GALK1-inhibitors, superoxide dismutase and enzyme replacement therapy to increase the activity of human GALT.

## Conclusion

In conclusion, the results of this pilot study suggest that neither arginine nor arginine aspartate (Asparten®) would be therapeutically effective in galactosemia patients homozygous for the c.563A > G (p.Q188R) mutation under the presented conditions. Nevertheless, these findings do not preclude that arginine could be successfully used in patients carrying other *GALT* mutations. Further studies are warranted to gain more insight in arginine’s effect on other *GALT* mutations.

## Patients and methods

### Patients

Four adult classic galactosemia patients being followed up in our expertise centrum for classic galactosemia carrying the p.Q188R mutation in homozygosity were included in the study (Table 1). Written informed consent was obtained from all patients participating in the study. The study was approved by the Medical Ethical Committee of the Maastricht University Medical Center + (METC). This study has been registered at https://clinicaltrials.gov (NCT03580122).

### Study design and intervention

This is a study with a pre-post single arm design. All patients received arginine in the commercially available form of arginine aspartate (Asparten®). This formula was chosen based on the fact that it is administered by oral route and it displays well-known pharmacokinetic and toxicological characteristics. It is commercialized since 1974 and no side effects have ever been described. Each ampoule of Asparten® contains 5 g of arginine aspartate. Following the screening and baseline assessments, patients received 5 g of arginine aspartate, 3 times a day (as recommended in Asparten®‘s Summary of Product Characteristics), corresponding to a daily dose of 15 g arginine aspartate. Duration of the intervention was 1 month (30 ± 5 days). Patients were asked to keep the empty ampoules in order to ascertain compliance to intervention.

### Diet

All patients remained on a galactose-restricted diet, which consists of eliminating all galactose- and lactose-containing dairy products from the diet (except for mature cheeses and caseinates); non-dairy sources of galactose, such as fruit and vegetables are allowed.

Patients were asked to keep a 3-day diary of their diet in the beginning and in the end of the study to be able to calculate the average arginine intake.

### Assessments

All patients were evaluated at baseline and after intervention. Primary outcome was whole body galactose oxidative capacity. Secondary outcomes included erythrocyte GALT activity, as well as urinary galactose and galactitol levels. Additionally, pre-prandial amino acid profile was also performed.

Baseline assessments were done immediately before the initiation of Asparten® supplementation, and after treatment assessments were done immediately after suspension of Asparten® supplementation.

### Primary outcome

Whole body galactose in vivo oxidative capacity was determined as previously described [17, 23]. Patients were administered 7 mg/kg [1-^13^C]-galactose tracer (0.039 mmol/kg, Cambridge Isotope Laboratories, Andover, USA) via a single bolus injection. Expired breath samples were collected into 10 mL Exetainer tubes (Labco limited, Lampeter, UK), which were filled directly from a mixing chamber in duplicate. A baseline breath sample was collected prior to [1-^13^C]-galactose injection (*t* = 0 min). During the first hour following injection, breath samples were collected at *t* = 5, 15, 30, 45 and 60 min. Thereafter, breath samples were collected at 30-min intervals until *t* = 360 min. Whole-body oxygen uptake (VO_2_) and carbon dioxide (VCO_2_) production were measured during three 60 min periods (*t* = 30–90, 150–210, and 270–330 min) using a ventilated hood system (Omnical, Maastricht University, Maastricht, the Netherlands). During the entire study period, subjects rested supine and were allowed to drink only water.

Expired breath samples were analyzed for ^13^C/^12^C ratio by gas chromatograph continuous flow isotope ratio mass spectrometry (Finnigan, Bremen, Germany). The isotopic enrichment was expressed as δ per mil difference between the ^13^C/^12^C ratio of the sample and a known laboratory reference standard [24].

The δ^13^C was then related to an international standard (PDB-1). The ^13^CO_2_ production from the oxidation of the infused [1-^13^C]-galactose tracer was subsequently calculated as:

$$ {\Pr}^{13}{\mathrm{CO}}_2=\frac{\left({\mathrm{TTR}}_{{\mathrm{CO}}_2}\ \mathrm{x}\ {\mathrm{VCO}}_2\right)}{(k)} $$

Where ${\mathrm{TTR}}_{{\mathrm{CO}}_2}$ is the breath ^13^C/^12^C ratio at a given time point, VCO_2_ is the carbon dioxide production (L∙min^− 1^), k is the volume of CO_2_ (22.4 L∙mol^− 1^). Total ^13^CO_2_ production represents a minimal estimate of the total amount of [1-^13^C]-galactose that was oxidized within the experimental period. In addition, the cumulative percent of the [1-^13^C]-galactose tracer eliminated as ^13^CO_2_ in expired air (CUMPCD) was calculated.

### Secondary outcomes

GALT enzymatic activity in RBC was performed as previously described [25]. Galactose and galactitol levels were measured in urine by GC/MS.

Amino acid profile was measured as described by Waterval et al. [26].

### Diet

Dietary intake of arginine was determined by a 3-day food diary pre and post intervention and calculated as mean percentage of the total protein intake. The amount of arginine was considered to be 5% of the total protein intake [27].

### Fibroblasts

Cultured fibroblasts from two wildtype and three classic galactosemic patients (Coriell GM1703, GM727 with p.Q188R/p.Q188R mutations and a Boston Children’s Hospital patient with p.Q188R/p.Q188P mutations) were grown in MEM media supplemented with 10% FBS at a 37 °C incubator with humidified atmosphere of 5% CO_2_. Arginine treatment was started by supplementing the growth media with 0, 0.1 mM or 1.0 mM of arginine (considered as day 0 of arginine treatment). On day 3 of the arginine treatment, cells were washed with ice cold PBS and pellets were harvested in cold PBS by scraping. The pellets were stored in − 80 °C until enzyme measurements. The GALT activity in cell lysates was measured using LC-MS/MS by a modified version of a method previously described for washed RBC [28]. Cell lysates were prepared by resuspending the pellets in 100–200 μL 0.5 M glycine buffer (pH 8.7) and sonicating for 15 s at level 1 (Sonic Dismembrator model 100, Fisher Scientific, USA) three times with one-minute intervals. The homogenate was then centrifuged at 15,800 g for 10 min at 4 °C (5402R, Eppendorf). Protein concentration was quantified using DC kit (Bio-RAD). AB Sciex QTrap 5500 mass spectrometer was used for quantification of the enzyme product. Arginine was measured in the cell culture supernatant by stable isotope dilution liquid chromatography tandem mass spectrometry (LC-MS/MS) using a ^15^N-L-Arginine internal standard (Cambridge Isotope Laboratories Inc., Tewksbury, MA, USA) with an Acquity™ Ultraperformance® liquid chromatography system coupled to a Quattro Premier tandem-quadrupole mass spectrometer (Waters Corporation, Milford, MA, USA).

### Recombinant protein analysis

Expression and purification of human GALT was performed as previously described [6]. Differential scanning fluorimetry (DSF) was performed in a 96-well plate using an Mx3005p RT-PCR machine (Stratagene) with excitation and emission filters of 492 and 610 nm, respectively. Each 20 μL reaction consisted of 2 μL as-purified recombinant protein (final concentration of 2 μM, wildtype or p.Q188R) and 2 μL arginine at various concentrations (100 μM to 10 mM) in DSF buffer (150 mM NaCl, 10 mM HEPES pH 7.5) to which 2 μL SYPROrange, diluted 500-fold in DSF buffer from the manufacturer’s stock (Invitrogen) was added. Galactose (10 mM) was used as an indicator of ligand binding (positive control). Fluorescence intensities were measured at each 1 °C temperature increase from 25 to 96 °C with a ramp rate of 3 °C/min. A thermal shift of 3 °C or more is considered to be significant, though interpretation varies, and some papers report a value > 1.5 °C as significant.

### Statistical analysis

Arginine aspartate’s effect in the primary and secondary outcomes was evaluated in IBM SPSS Statistics 23, using a paired t-test (double-sided) with a natural correction for non-normal populations. A *p* value less than 0.05 was considered statistically significant.

## Additional files

Additional file 1: **Figure S1.** Expired breath 13CO2 enrichments. Expired breath 13CO2 enrichments of the four galactosemia patients, before and after arginine aspartate supplementation. Results are expressed as mean ± SEM. (PDF 95 kb)

Additional file 2: **Table S1.** Concentration of arginine in the cell culture supernatant. The concentration of arginine was measured at day 0 and 3 of incubation in two wildtype (Control line 1 and 2) and two classic galactosemic fibroblasts derived from two p.Q188R/p.Q188R patients (cell lines CG1 and CG2) in the absence and in the presence of supplemental arginine (0.1 mM and 1 mM arginine). Results are expressed as μM. (PDF 16 kb)

## Abbreviations

CG: Classic galactosemia
CUMPCD: Cumulative percent of the dose
DSF: Differential scanning fluorimetry
GALT: Galactose-1-phosphate uridylyltransferase
METC: Medical Ethical Committee, In Dutch: Medisch Ethische Toetsingscommissie
NO: Nitric oxide
PDHc: Pyruvate dehydrogenase deficiency
RBC: Red blood cells
VCO2: Carbon dioxide production
VO2: Whole body oxygen uptake
