# Supplementary material for: Arginine does not rescue p.Q188R mutation deleterious effect in classic galactosemia
Source: Orphanet J Rare Dis. 2018 Nov 26;13:212. doi: 10.1186/s13023-018-0954-8 (PMC6260575; doi:10.1186/s13023-018-0954-8)
Supplement: Supplementary file 1 — Figure S1. Expired breath 13CO2 enrichments. Expired breath 13CO2 enrichments of the four galactosemia patients, before and after arginine aspartate supplementation. Results are expressed as mean ± SEM. (PDF 95 kb) [file 13023_2018_954_MOESM1_ESM.pdf]

Supplementary Figure S1

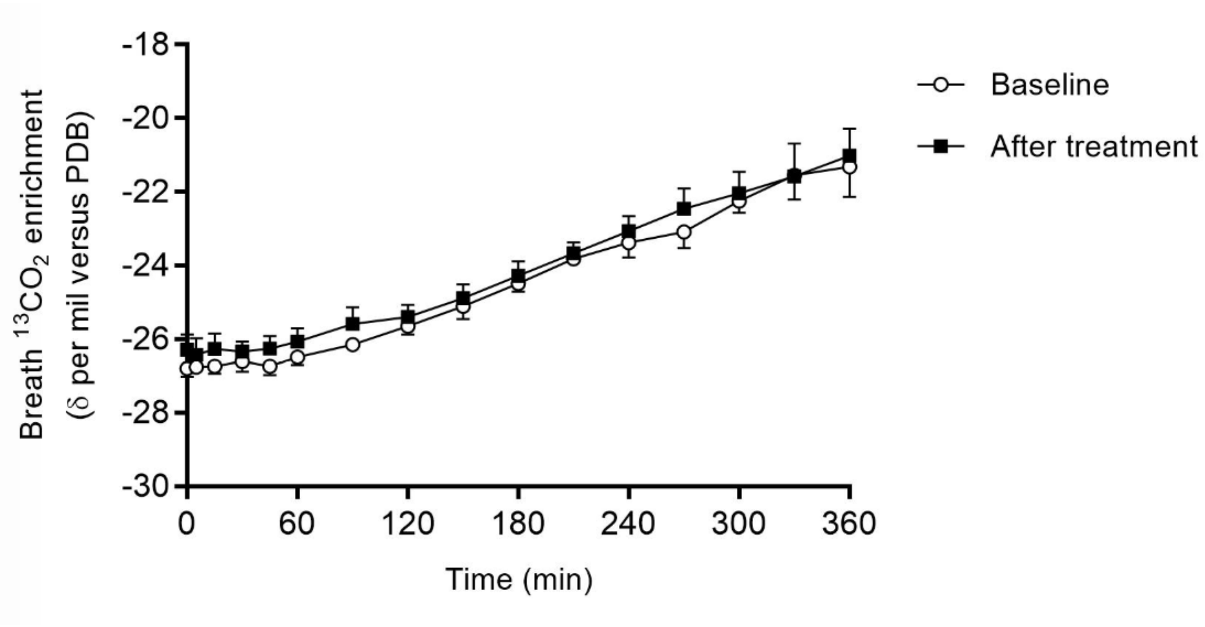

Supplementary Figure S1. Expired breath $^{13}CO_2$ enrichments. Expired breath $^{13}CO_2$ enrichments of the four galactosemia patients, before and after arginine aspartate supplementation. Results are expressed as mean ± SEM.
