# Supplementary material for: Arginine does not rescue p.Q188R mutation deleterious effect in classic galactosemia
Source: Orphanet J Rare Dis. 2018 Nov 26;13:212. doi: 10.1186/s13023-018-0954-8 (PMC6260575; doi:10.1186/s13023-018-0954-8)
Supplement: Supplementary file 2 — Table S1. Concentration of arginine in the cell culture supernatant. The concentration of arginine was measured at day 0 and 3 of incubation in two wildtype (Control line 1 and 2) and two classic galactosemic fibroblasts derived from two p.Q188R/p.Q188R patients (cell lines CG1 and CG2) in the absence and in the presence of supplemental arginine (0.1 mM and 1 mM arginine). Results are expressed as μM. (PDF 16 kb) [file 13023_2018_954_MOESM2_ESM.pdf]

Supplementary Table S1

Supplementary Table S1. Concentration of arginine in the cell culture supernatant. The concentration of arginine was measured at day 0 and 3 of incubation in two wildtype (Control line 1 and 2) and two classic galactosemic fibroblasts derived from two p.Q188R/p.Q188R patients (cell lines CG1 and CG2) in the absence and in the presence of supplemental arginine (0.1 mM and 1 mM arginine). Results are expressed as μM.

	**0 mM arginine**	**0.1 mM arginine**	**1 mM arginine**
**Media at day 0**	531	657	1548
**Media at day 3 - CG line 1**	486	587	1374
**Media at day 3 - CG line 2**	547	606	1562
**Media at day 0**	484	524	1346
**Media at day 3 - Control line 1**	475	550	1339
**Media at day 3 - Control line 2**	463	530	1317
